# Drug Delivery Application of Functional Nanomaterials Synthesized Using Natural Sources

**DOI:** 10.3390/jfb14080426

**Published:** 2023-08-15

**Authors:** Mekala Veerapandian, Subramaniyan Ramasundaram, Peter Jerome, Gayathri Chellasamy, Saravanan Govindaraju, Kyusik Yun, Tae Hwan Oh

**Affiliations:** 1 Department of Bionanotechnology, Gachon University, Soengnam 13120, Republic of Korea; mekalaveerapandian@gmail.com (M.V.); gayathri.chellasamy@gmail.com (G.C.); biovijaysaran@gmail.com (S.G.); 2 School of Chemical Engineering, Yeungnam University, Gyeongsan 38436, Republic of Korea; ramasundaram79@hotmail.com (S.R.); jeromepeter7688@gmail.com (P.J.)

**Keywords:** natural sources, drug delivery system: metallic nanoparticles, polymeric nanomaterials, carbon dots, mucilage and gums, exosomes

## Abstract

Nanomaterials (NMs) synthesized from natural sources have been attracting greater attention, due to their intrinsic advantages including biocompatibility, stimuli-responsive property, nontoxicity, cost-effectiveness, and non-immunogenic characteristics in the biological environment. Among various biomedical applications, a breakthrough has been achieved in the development of drug delivery systems (DDS). Biocompatibility is necessary for treating a disease safely without any adverse effects. Some components in DDS respond to the physiological environment, such as pH, temperature, and functional group at the target, which facilitates targeted drug release. NM-based DDS is being applied for treating cancer, arthritis, cardiovascular diseases, and dermal and ophthalmic diseases. Metal nanomaterials and carbon quantum dots are synthesized and stabilized using functional molecules extracted from natural sources. Polymers, mucilage and gums, exosomes, and molecules with biological activities are directly derived from natural sources. In DDS, these functional components have been used as drug carriers, imaging agents, targeting moieties, and super disintegrants. Plant extracts, biowaste, biomass, and microorganisms have been used as the natural source for obtaining these NMs. This review highlights the natural sources, synthesis, and application of metallic materials, polymeric materials, carbon dots, mucilage and gums, and exosomes in DDS. Aside from that, challenges and future perspectives on using natural resources for DDS are also discussed.

## 1. Introduction

**Figure 1:**
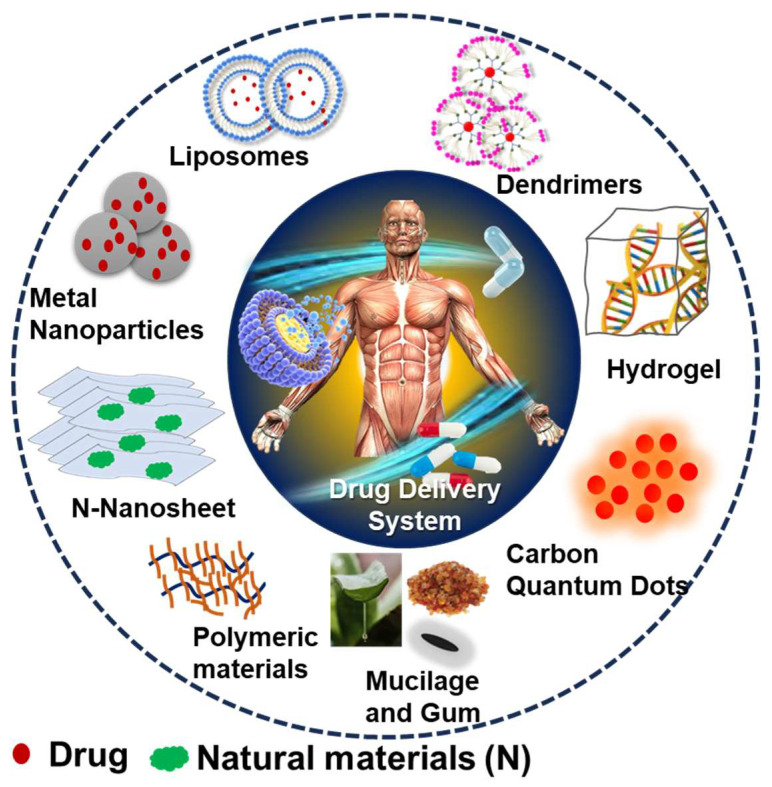
Depiction of the different shapes and structural NMs toward drug delivery systems.

**Figure 2:**
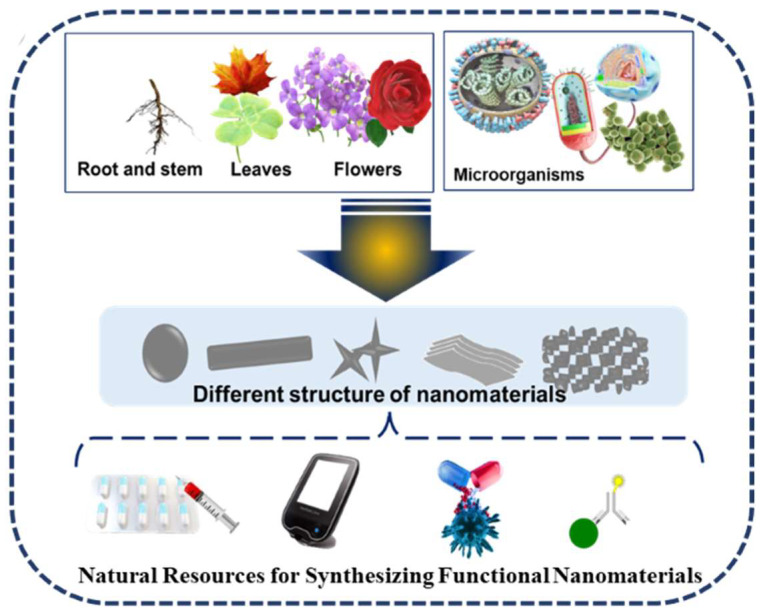
Plant parts and microorganisms used in the synthesis of functional nanomaterials toward biomedical application.

Nano drug carriers are important for addressing the delivery of drugs with poor solubility, high toxicity, and less bioavailability. Various nanocarriers synthesized from natural resources such as organic, metallic, and polymeric NMs, including liposomes, exomes, dendrimers, and micelles, are formulated for target DDS. A depiction of the different shapes and types of the natural sources of NMs toward DDS is shown in Figure 1. As synthesized nanomaterials have different shapes such as nanosheets, nanopins, nanotubes, etc., and different sizes, they are being used as DDS for various diseases like cancer, brain, heart, skin, and bone diseases. Their properties, such as size, surface charge, shape, and hydrophobicity, also enhance the bioactivity of nanomaterials. As a substitute for synthetic materials, NMs prepared using natural sources can be incorporated in DDS for performing various functions such as carrier, imaging, stimuli responsive moiety, and super disintegrant. The natural resource-based synthesis of NMs started from the 1900s [1,2,3]. Mostly, phenols, alcohols, saponins, alkaloids, and terpenes containing natural resources have been used. Various types of natural sources such as plant extracts, roots, stems, flowers, and microorganisms have been used for the synthesis of the above-mentioned nanomaterials [4]. In particular, the plant extracts, activated carbon, biomass, biowaste, fungi, algae, bacteria, chitosan, gelatin, fibrin, cellulose, and chitin are some of the natural sources used for obtaining these NMs. The natural resources used for the synthesis and morphology of NMs for various biomedical applications is presented in Figure 2. The use of natural resources improves the safety of the final product. The use of natural resources for the synthesis offers renewable materials based on green technology and reduces the hazardous components, such as toxic chemicals as reducing agents, surfactants, and the risks in post effluent treatments.

Unlike synthetic materials, the components derived from natural resources or sustainable biological systems do not possess potential side effects or inherent toxicity. The functional NMs from renewable natural resources offer biocompatibility, versatile surface functionalities, and biodegradability. The improvement in biocompatibility increases the safety margin of drugs as they prevent side effects and drug rejection [5]. Biocompatible and biodegradable functional components can minimize the toxicity of nonbiodegradable residual parts used in DDS. DDS based on natural anticancer agents exhibits improved bioavailability, biodistribution, therapeutic activity, and stability [6,7]. For example, naturally derived tricalcium phosphate (TCP) shows high protein encapsulation efficiency than synthetic TCP with an identical Ca/P ratio [8]. These green drug carriers were found to increase the therapeutic activity of anticancer drugs by facilitating the controlled release and increasing the extent of targeting [9].

**Figure 3:**
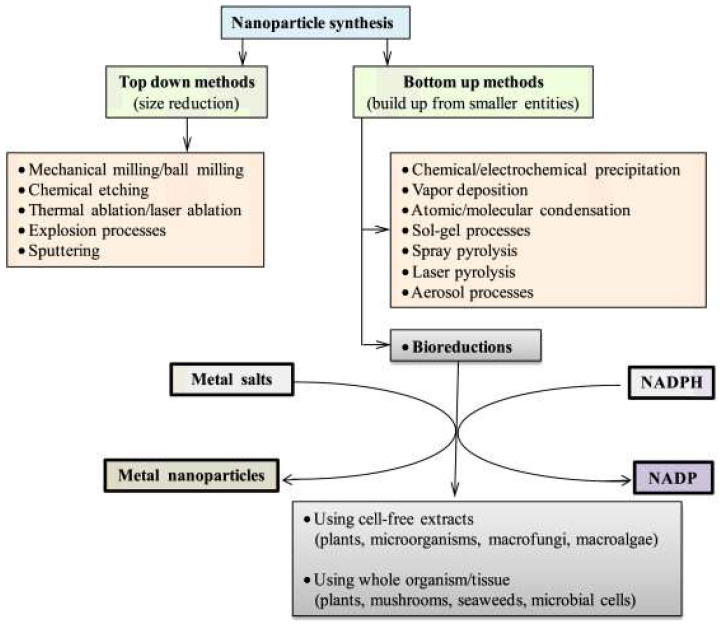
Bottom-up and top-down synthesis methodologies used in natural source-based functional nanomaterials synthesis (adopted with permission from Ref. [3]).

The naturally derived molecules have been used in the synthesis of metal-based NMs due to their reducing and stabilizing properties. The molecules with reducing and capping properties are used for the single-step synthesis of metal- and metal oxide-based NMs, especially nanoparticles (NPs). Gold (Ag), copper (Cu), silver (Ag), and iron oxide (Fe_2_O_3_) are used in typical DDS. The metal-based NMs are widely used in DDS as a therapeutic agent and imaging agent for diagnosis. Certain phytoconstituents, phenolics, enzymes, vitamins, proteins, polysaccharides, and other biomolecules possess the natural behavior of reduction and capping. Once the stock of natural extract is obtained, the natural resource-based synthesis can be completed in a single step that is economic and consumes less time than the chemical methods [3,10]. Bottom-up and top-down synthesis methodologies based on the natural sources are depicted in Figure 3.

In DDS, naturally obtained polymers are used as a carrier, stimuli responsive moiety, target specific biding interface, and stealth coating. The molecules with biological activity, such as anticancer, antimicrobial, and anticoagulant activity, was used as therapeutic agents or natural drugs. Plant- or microbe-derived poly(saccharides) and poly(phenols) can act as a mechanical support ligand for linking and delivering the drug [11]. Natural polymers are ecofriendly, nonirritant, biodegradable, biocompatible, cheap, and easily available. Chitosan acquired from animals, plants, seaweed, and microorganisms is an excellent example of a naturally derived biopolymeric drug carrier. In addition to that, chitosan can reduce and stabilize metal salts. The amino group in chitosan can bind the drugs and biological molecules via a non-covalent interaction sensitive to pH, temperature, and molecules using competitive interactions [12,13,14].

Carbon quantum dots (CDs) have functional groups such as hydroxyl, epoxy, carboxyl, and amino on their surface and these surface moieties can bind with inorganic or organic functional groups endowing both covalent and non-covalent interactions. CDs are capable of molecular recognition. When irradiated under ultraviolet radiation (UV, λ = 370 nm), CDs exhibit fluorescence. In DDS, CDs function as performance components for imaging, targeting, drug delivery, and photodynamic therapy. The hydrothermal carbonization of natural resources and waste spanning from rice husk to food waste have been used as the raw materials for preparing natural carbon-based CDs (NCDs) [15,16]. The plant-derived mucilage and gums (MGs) are also used as the drug encapsulation matrix and environment-responsive drug release agents. MGs are a bio macromolecular assembly. MGs have been traditionally used as the excipient and bioadhesive in drug formulations. Excipients are the non-active constituents in the drug formulation, such as the protective coating, bulking agent, binders, diluents, coloring material, and flavoring agents. MGs exhibit mucoadhesive behavior and are stable, non-toxic, naturally available in abundance, and undergoes less regulatory issues than the synthetic polymers [17,18].

Exosomes are small endosomal, naturally nanosized (30–150 nm) extracellular vesicular structures that are enclosed by a lipid bilayer membrane. Exosomes are mostly present in eukaryotic cells and released from the cells during several physiological processes. Despite their early discovery in 1980 [19], most often, due to its secretion from the cells via several metabolic processes resulting from cell damage, cellular homeostasis, etc., they were initially distinguished as “cellular wastes” [20]. Later, exosomes emerged as “superior functional nanovehicles” due to reasons such as the significance of these membrane-enclosed extracellular vehicles (EVs) in establishing intercellular communication, their ability to penetrate the bio membranes and escape during metabolic destruction, the lack of undesired accumulation, and their innate biocompatibility [21,22]. As far as DDS is concerned, the source, processing, synthesizing methodology, and functionalization of the above functional NMs are the most important. This review discusses the important efforts taken towards the development of DDS based on metallic materials, polymeric materials, NCDs, MGs, and exosomes derived or synthesized using natural resources.

## 2. Natural Source-Synthesized Au and Ag NPs in Drug Delivery Systems

Flavonoids also play a main role in the NMs’ synthesis as a reducing agent and in the chelation of metal ions. Flavonoids from the *Ocimum basilicum* and *Mangifera indica* leaf were used for the synthesis of Ag NPs and plays a main role in the reduction in Ag ions to Ag NPs. The other most potential flavonoid, quercetin, also acts as a strong chelating agent due to its carbonyl and hydroxyl groups. These kinds of biological molecules have important roles in the synthesis of NMs to inhibit its aggregation and enhance the stability and solubility in biomedical applications. Vitamin C is also a well-known reducing agent, as it can reduce the metal ions into metal NPs in an aqueous medium. Vitamin C is used to reduce the iron hydroxide into iron NPs during the hydrothermal process. The other vitamins, like B and E, are also used to reduce the metal ions into metal NPs [23,24,25,26,27]. Biosynthesized metallic NPs possess various biological activities such as anti-cancer, anti-inflammatory, and antimicrobial activities. When combined with drugs, these biological activities are used for addressing drug resistance.

The surface plasmonic properties of metallic NPs are useful for tracking the biodistribution of drugs via imaging, photothermal therapy, and theranostic applications. Au and Ag NPs have been widely used, as they can be conjugated with various biologically relevant molecules including oligosaccharides and proteins. These molecules are useful for receptor-sensitive targeting of the pathobiological site to be treated. The use of chemical reducing agents such as sodium borohydride, hydrazine hydrate, phenyl hydrazine, and sodium citrate are considered as toxic to humans. These reducing agents require the use of surfactants for stabilization. In order to combat the issues affecting the environment and addressing the toxicity to human health posed by biomedically relevant metallic NPs, biosynthesis methodologies capable of yielding eco-friendly and biocompatible products have been preferred to use in DDS [10]. During biosynthesis, the plant- and microorganism-derived reducing agents have also been used as stabilizing/capping agents. Au and Ag NPs synthesized using plant extract and microbe-derived proteins were reported as efficient to be used in DDS. The selection of natural resource with a high abundance of constituents capable of reducing/capping metallic NPs assures the stability required during their application in DDS.

Kim et al. reported a strategy for preparing NPs of Au, Ag, cadmium selenide (CdSe), and europium selenide (EuSe) inside an alginate gel matrix by using the cell lysate obtained from a recombinant Escherichia coli strain. The alginate gel is used as a bioreactor. The cell lysate contains metal binding proteins, Rabidopsis thaliana phytochelatin synthase (AtPCS), and *Pseudomonas putida* metallothionein (PpMT). When exposed to UV irradiation, the metal precursor reduces to form metal nanoparticles due to the photochemical catalyzing agent and sensitization of the metal precursor. The thiol groups present in the proteins play a pivotal role in the synthesis of metallic NPs. While the metal NPs are used as an imaging agent, the alginate gel is used as a bioreactor as well as a carrier for encapsulating dual drugs, doxorubicin (DOX), and Rifampicin (RIF). The drug release was pH dependent. RIF, encapsulated with CdSe@gel and EuSe@gel, was not released under acidic pH and the release was increased above pH 7.4. The release of RIF was completed within ~250 min. At pH 7.4, DOX release continued up to ∼400 min. The electrostatic interaction between alginate and drugs played a decisive role in the drug release [28].

Au NPs (43.0 ± 2.2 nm) synthesized using the aqueous extract of walnut tree (Juglans regia) bark was used for the controlled release of zonisamide (ZNS, 1,2-benzisoxazole-3-methanesulfonamide). ZNS is an antiepileptic drug used for treating psychiatric and neurological diseases (Parkinson’s disease and epilepsy). The polyphenolic compounds present in walnut bark can reduce and cap the metal salts in metal NPs. The ZNM-Au NP complex was prepared via simple solution blending of ZNS and Au NPs. When tested in vitro, ZNS-Au NPs exhibited a controlled release of ZNS. After 10 days, 31% of ZSM was released, whereas an 81% release was noticed when free ZNS was used. The ZNS-Au NP complex also hindered the growth of CTX TNA2 cells. Au NPs were used as a carrier for ZNS, and this complex was proposed as useful in treating acute spinal cord injury [29].

The magnetic, anti-oxidant, antibacterial, and anticandidal properties of Fe_3_O_4_ NPs (37.8 nm) are widely utilized in theranostic imaging and antibiotic and anticandidal drugs formulations. Patra et al. synthesized Fe_3_O_4_ NPs using the aqueous extract of corn ear leaves. The major chemical constituent (37 to 64%) present in corn leaves is phytol (3,7,11,15-tetramethyl2-hexadecen-1-ol). The Fe_3_O_4_ NPs exhibited proteasome inhibitory, antioxidant, and anticandidal activities. Proteasome inhibitory activity was expected useful in cancer treatment and drug delivery. The Fe_3_O_4_ NPs were considered useful in drug delivery systems meant for addressing the drug resistance and toxicity [30]. Epigallocatechin gallate (EGCG), the polyphenol-type compound found in green tea, was used for synthesizing the flower-shaped Au nanoclusters (50 nm, Au NCs). EGCG is most abundant in green tea and used as both a reducing and capping agent. Au NCs were stable for 6 weeks and used as a carrier for methotrexate and DOX. These drugs were conjugated with Au NCs via a cysteine bridge. This dual drug conjugate was used for tumor-targeted drug delivery. When tested with HeLa cells in both in vivo and in vitro conditions, these conjugates were entered into the cell. The folic acid receptor’s overexpressed tumor cells can be targeted by this conjugate. The efficient cellular uptake and sustained drug release increased the bioavailability (acting time) of the drugs [31].

Inorganic NPs can also be synthesized via the metabolism of living microbes, including bacteria, fungi, and yeast. When the metal ions were taken by these organisms, insoluble precipitates were formed via bioreduction caused by the metal-binding protein secreted by this microbe when maintaining homeostasis. Similar to Kim et al., PpMT and AtPCS were used for the in vitro biosynthesis of FeSe NPs under UV irradiation. The FeSe NPs were conjugated with poly-γ-glutamic acid (PGA). This conjugate was used as a carrier for encapsulating DOX, a nonselective anti-cancer drug whose continuous consumption leads to the inherent risk of long-term side effects in the heart, kidney, liver, and brain. So, the targeted and controlled release of DOX is necessary to prevent any side effects. PGA is a non-toxic and naturally occurring peptide capable of exhibiting pH-dependent conformational change. Under acidic pH, PGA can retain the drugs loaded inside the conjugate and quickly releases the drug at pH 6–7. The use of FeSe NPs allowed for the mapping of the biodistribution of the FeSe NPs-PGA-DOX conjugate over time in mice, and also confirmed the delivery of drugs to the target [32].

An aqueous extract of meadow grass, *Poe annua* (*P. annua*), was used for the synthesis of Ag NPs (36 to 43 nm). The flavonoids in *P. annua* can be used as a non-toxic, eco-friendly, and cost-effective reducing agent for the synthesis of Ag NPs. The Ag NPs were coated with flavonoids, phenols, and alkaloids present in *P. annua*. These Ag NPs were used as a carrier for delivering anticancer biomedicine derived from Euphorbia dracunculiodes Lam (EDL). EDL is a medicinal plant used in Indian and Chinese traditional medicine and applied for treating neurodegenerative diseases, cardiovascular diseases, and diabetes mellitus. EDL is a source of phenolic compounds and triterpenoids. The conjugate of Ag NPs and EDL were prepared and stabilized with starch coating. When orally administered to Sprague Dawley rats, there was no significant cytotoxicity found even after 7 days. This conjugate exhibited a good biocompatibility to healthy cells but targeted cancer cells. The combination of biosynthesized Ag NPs and plant-derived anticancer medicine has proven to be a promising anti-cancer nanomedicine [33]. Furthermore, the green-synthesized metal-based NPs have a certain limitation in the parameters (temperature and pH) and stability. The obtained NPs are less stable in nature as compared to chemically-synthesized NPs. NPs ensure stabilization in two ways: electrostatic repulsion and steric stabilization. For the electrostatic repulsion, the extract should have low ionic strength, which results in a more dispersed double layer. In the case of high ionic strength, which leads to the van der Waals interaction. Secondly, the steric stabilization provides the surface barrier [34,35]. For large scale productions, it is necessary to overcome these types of limitations and develop the green synthesis of metal-based nanoparticles.

## 3. Natural Source-Derived Polymeric Nanomaterials in Drug Delivery Systems

**Figure 4:**
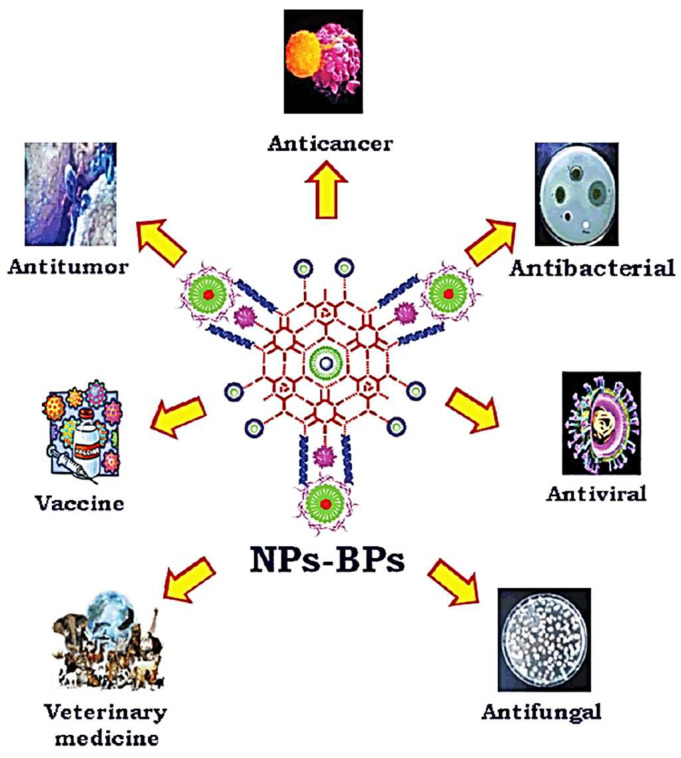
Drug delivery applications of biopolymer modified with nanoparticles (adopted with permission from Ref. [6]).

The surface of NMs incorporated with DDS is very important as they directly contact the body fluids and organs. The hydrophilic polymer-coated NMs stay a long time in blood circulation and performs site-specific delivery upon rupture in their interactions with drugs by surrounding the pH and enzymes. Typically, naturally occurring polymers such as poly(peptides), poly (amino acids), poly(saccharides), starch, collagen, alginate, gelatin, elastin, chitosan, and cellulose are known to be degraded under enzymatic conditions. These natural polymers can be obtained from both animal and plant sources. For example, cellulose can be derived from both plants and bacteria. Plants derivatives or bacteria are the main sources for the nanocellulose materials (Nanosize cellulose fibers or nanocrystal). The bacteria species, such as *Gluconacetobacter*, *Agrobacter*. and *Sarcina*, are known to be capable of producing highly pure and water-retaining cellulose. Due to the difficulties in preparing NMs, these natural polymers were cross-linked at the surface of nanosized drugs and imaging agents. A schematic illustration of the application of biopolymer modified with nanoparticles in DDS is shown in Figure 4. The natural polymer-modified surface is used for forming protective, biocompatible, stimuli responsive surfaces [36].

The biopolymer, Kappa carrageenan (KC), extracted from microalgae was conjugated with ZnO NPs and used as a biofilm-based drug carrier. KC is water soluble and contains sulfated D-galactan. The KC-ZnO conjugate is a non-toxic and eco-friendly “nano-anti super bug drug”, used as a nano-antibiotic for treating disease infections caused by Methicillin-resistant Staphylococcus aureus (MRSA). KC functions as a capping agent and plays a major role in inhibiting bacterial cell growth. KC also reduces the hydrophobicity of the bacterial cell surface. As a result, the KC-ZnO nonocomposite was applied to the in vitro anti-inflammatory using the embryonic fibroblast cell line derived from the NIH3T3 mouse. About 82% of anti-inflammatory activity was noticed [37]. Green synthesis of the hydrolyzed starch–chitoson (HS-C-NC) composite were applied as DDS against the Gram-negative bacteria. This combination of two biopolymers loaded with the antibiotic exhibited good results than the individual biopolymer. Ciprofloxacin loaded with HS-C-NC was prepared via an ionic exchange method. The studies carried out in the Gram-negative bacterial strains, *Enterobactor aerogenes* and *Pseudomonas aeruginosa*, demonstrated that the minimum inhibition concentration (MIC) of the Ciprofloxacin-loaded HS-C-NC were 62.5 and 125 µg/mL, respectively. The in vitro drug release study showed a 86% drug release was attained after 20 h at pH 1.5, and it was 33% at pH-7.5. The intact structure and high inhibition activity were considered as the reason for the good performance of HS-C-NC [38].

Almaida, et al. investigated DDS based on the natural rubber latex (NRL) membrane with various pore sizes such as 2000, 6000, and 10,000 pore/cm^2^. The drug loading capacity of NRL was evaluated via swelling studies, where the swelling rate of NRL reached its maximum (1.07 ± 0.03 g/g). NRL is used as a solid matrix drug delivery as it enhances the wound healing process. The NRL membrane loaded with ciprofloxacin showed the kinetics of drug release dependence on the pore size linearly increasing at the final concentration. There was an interaction between ciprofloxacin and NRL via hydrogen bonding. The drug release was determined using an ultrafast laser micromachining process [39]. Molecularly-imprinted polymer (Chitosan) (MIP) was also used in DDS. MIP has high chemical and mechanical strength, high absorption, selectivity, and storage capability of a prolonged lifetime without loss of the target molecule. MIP was used as a carrier for riboflavin, a drug used for stabilizing the vitamin B_2_ in our body. The drug release was determined via the Higuchi model. Riboflavin loaded in the polymer showed a good drug releasing efficiency (55.07%), with a 1:20 ratio of the NaCl media. This amount is equal to the vitamin B_2_ requirement in humans for 5 days [40].

Chitosan is majorly used in DDS because it is a cationic biopolymer, so the drug was easily loaded and served as a good carrier. The functionalization of NPs derived from ginger-derived chitosan (C-GDNC) was used for the controlled release of 5-amino salicylic acid (5-ASA), the drug used for treating inflammatory bowel diseases (IBD). They observed that the controlled release of the drug was more than 50% at pH 6.8. The polysaccharides play a vital role in drug delivery as well as has a self-healing potential against IBD [41]. Lignin-based hallow nanospheres were synthesized using the efficient fast vacuum evaporation process and are used as a carrier for Ibuprofen. This DDS exhibited excellent pH responsive drug delivery because of the repulsive force generated by the deprotonated carboxyl and hydroxyl groups present in lignin. At a neutral pH, a 90% drug release was noticed within 2–3 h, and at pH 1.8, only 18% of drug was released [42].

The biopolymer, pectin, was synthesized from plant cells consisting of rhamnogalacturonan I (RG-I), galacturonan, and rhamnogalacturonan II (RG-II) proanthocyanidine. Gunter et al. green-synthesized pectin from a callus culture encapsulated with ZnO hydrogel beads and used it for developing and delivering drugs in the gastrointestinal tract. The grape seed extract (GSE) delivery system consists of proanthocyanidines and phenol hydroxyl groups, where both have great potential for treating colon inflammation. But, the phenol group present in the GSE has poor stability when it reaches the gastrointestinal tract. When combined with hydrogels, the controlled release of GSE was achieved at pH 2.3 and 5.3. GSE-ZnO hydrogel was found to be promising in DDS for treating colon inflammation [43]. Hyaluronic acid (HA) is anionic polymer and also known as polysaccharides. HA, when combined with polyethyleneimine (PEI) to form nanogels (HA-PEI- NG), is applied for the controlled drug delivery in cancer treatment. In this mechanism, the hyaluronan receptor was involved in the binding process of nanogel, and there was no other surfactant used for drug binding with HA-PEI-NG. The NG was loaded with DOX. The high DOX encapsulation efficiency (83.4%) was noticed. The controlled drug release at the targeting site of the CD44 cell rendered a good therapeutic effect. The drug releasing efficiency in three different media (acidic, neutral, and alkaline) was determined via a drug diffusion mechanism, where the drug releasing efficiencies were 85%, 93%, and 97%, respectively [44]. Based on the literature survey, chitosan played a vital role in DDS due to being less toxic in nature compared to the synthetic polymers. Finally, the biopolymers provided good biomedical application especially in DDS because of their loading and nontoxic behavior. But, it is still necessary to investigate issues associated with stability and long-term-effect-related clinical studies.

## 4. Natural Source-Derived Carbon Dots in Drug Delivery Systems

**Figure 5:**
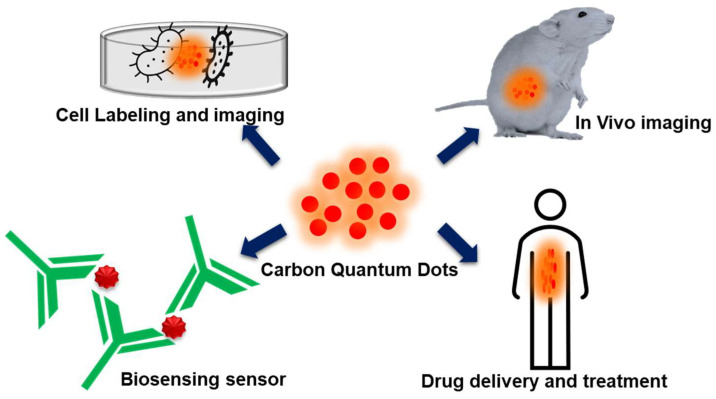
Drug delivery applications of natural resource-derived carbon quantum dots.

NCDs derived from natural sources have gained immense attention for developing DDS. NCDs are synthesized from natural products such as plant extracts, fruit juice, spices, milk, egg yolk oil, lotus root, honey, and food waste. NCDs are eco-friendly, water soluble, biocompatible, and available with diverse functional groups on the surfaces of hydroxyl, carboxyl, and thiol groups. The functional groups of NCDs can easily bind to the surface of metallic, polymeric, and biological molecules. Naturally synthesized CDs have good fluorescent properties and are used for drug delivery and bioimaging applications. NCDs have been used as a tracing probe, photo-activated antioxidant, and therapeutic and neurodegenerative agents [45,46]. Figure 5 depicts the application of NCDs in DDS. Iqra et al. developed an NCD-capped AgO/ZnO nanocomposite using banana juice as source for NCDs and the AgO/ZnO NPs were synthesized from *Fiscus carica* leaf extracts as a reducing agent to carry epirubicin for the chemotherapeutic drug delivery system to the breast cancer cells. This composite DDS inhibited 80% of cell viability, and 90% of cell viability was inhibited at a concentration of 50.0 µM, due to the electron acceptor and transporter property of NCD. When the nanocarrier is used in the acidic medium for the drug release, the cell viability decreased by 54.64% [47]. The nitrogen-functionalized NCD was used as a drug nanocarrier, as Morteza et al. prepared nitrogen-doped NCD and loaded the drug, gemcitabine (GC). The size of the nanocarrier is small, so it can easily enter and penetrate the cell membrane. When compared with a prepared NCD, the nitrogen-doped NCD exhibited a better anticancer effect [48]. NCD synthesized from pasteurized milk via the hydrothermal method served as a multi-functionalized hetero atom CD. These NCDs were used for encapsulating Lisionopril. When tested with HeLa cells, a controlled drug release was noticed at pH 5.2, 6.2, and 7.4. This composite shows as non-toxic to the Hela cells and can be used as effective DDS for treating hypertension and renal diseases [49]. Fluorescent quantum dots were synthesized via the hydrothermal synthesis method from mulberry leaves (*Morus alpha* L.). These NCDs were encapsulated with Lycorine, the anticancer drug, and the cytotoxicity study was performed with LX-2 and HepG2 cell lines. The cell biocompatibility study was performed with LX-2 cell lines. With a concentration up to 500 μg/, the NCDs showed good compatibility, whereas free drug biocompatibility was only up to 12 μg/mL. The NCD-Lycorine composite exhibited high anticancer activity to the HepG2 cell lines than the free drug. Lycorine-CDs can be useful to apply to intracellular imaging and target drug delivery to the cancer cells simultaneously. [50]. Hydroxyapatite-based NCDs (CD-HAP) were hydrothermally synthesized using a biowaste precursor under four different heating temperatures, 150, 170, 190, and 210 °C. The NCDs synthesized at 190 °C delivered high fluorescence and were used as a carrier for Acetaminophen, a common antibiotic. It has the highest loading capacity of 48.5% to the CD-HAP-40 and the drug releases slowed in CD-HAP-40 composite [51]. An aqueous root extract from Korean red ginseng was also used as a natural source for obtaining NCDs via microwave heating and were loaded with the drug, rutin (Ru-CDs). These NCDs were useful as both a drug carrier and imaging agent, emitting multicolor fluorescence and exhibiting reactive oxygen species scavenging in both an extra- and intra-cellular environment. The cytotoxicity study was performed with the NKE cell lines, and the Ru-CDs showed less toxicity at 100 μg/mL. Antibacterial activity was also performed against both the Gram-negative and Gram-positive bacteria under the light expose. It damaged produced more reactive oxygen species and also damaged the DNA in the bacteria [52]. To this extent, the QDs contain only carbon on one hand, and on the other hand, it did not have any toxic or heavy metals. Due to this property, it would be applied in DDS, imaging, and also in cancer therapy. But, there are still some issues related to reproducibility and clinical usage. The issues related to reproducibility should be rectified and more studies are needed that are related to QDs with cells and in vivo studies.

## 5. Natural Source-Derived Mucilage and Gums in Drug Delivery Systems

**Figure 6:**
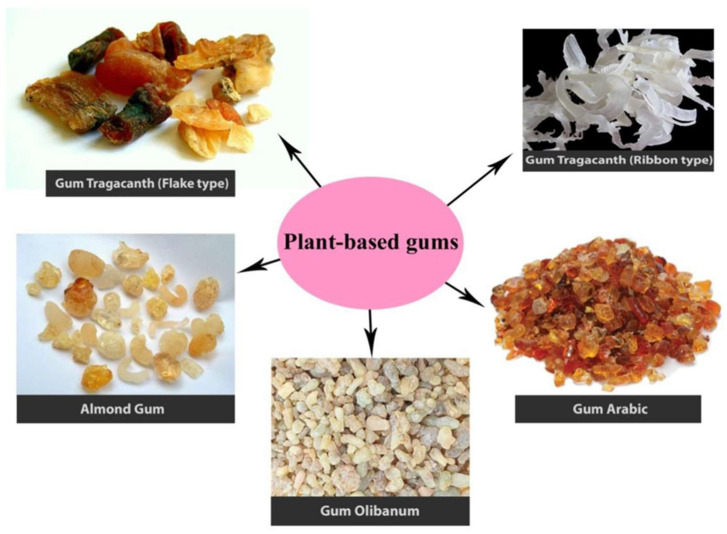
Mucilage and gums derived from various plant sources (adopted with permission from Ref. [54]).

MGs are naturally occurring, high molecular weight (~200,000) polyuronides with the units of sugar and uronic acid. They are the physiological product of plant metabolism. MGs are produced within the cell, deposited on its walls, and serve as a food reserve and membrane thickener. MGs function as a medium of water storage and aid in the germination of seeds. Figure 6 illustrates the various MGs derived from plant sources. Mucilage and gums (MGs) have been derived from animals, plants, microorganisms, and seaweed. MGs are more abundant in plants than in animals. The gums derived from herbal plants were applied as emulsifying, binding, thickening, suspending, stabilizing, and binding agents and sustained the drug release. Non-ionic (amylose, arabinans, and cellulose) and anionic (arabic, agar, algin, and pectic acid) MGs are available. MGs were extracted via heating and solvent extraction methods. Due to its sticky and mucoadhesive nature, MGs found an application in DDS. Additionally, the compounds present in the gums, named resins and tannins, provide a controlled drug release and inhibitory properties. The highly viscous and gradual swelling-induced slow aqueous solubility behavior of MGS increases the residence time of drug loading on the biological surface, such as ocular and gastrointestinal tracts; thereby, MGs are considered as the suitable carrier matrix for prolonged or sustained drug release. The constant contact established between the biological surface and DDS minimizes the drug resistance. Some of the important DDS-based MGs are discussed in this section [18,53].

The gum obtained from *Hakea gibbosa* (HG) was used for formulating mucoadhesive and sustained the release Buccal tablets. The chlorpheniramine maleate (CPM) was used as a model drug. The tablets without HG gum released 90% CPM within 14 min, whereas the tablets with 2, 12, 22, and 32 mg of HG gum exhibited a 90% CPM release after 48, 120, 330, and 405 min, respectively. The slow relaxation of the hydrated HG gum resulted in a sustained drug release. Aside from that, the excellent mucoadhesive properties for HG gum formulation and its mucoadhesive strength can be modulated by varying its quantity [54]. Galactomannan gum, derived from the seeds of *Mimosa scabrella*, was used for the controlled release of theophylline. This drug is used for treating asthma and chronic obstructive pulmonary disease. The in vitro drug release studies indicated the decrease in drug release in response to the increase in gum concentration. The formulation with 25% w/w of the gums exhibited an excessive sustained release. An erosion-type drug release was noticed with the tablets with lower gum concentration while high gum content led to fast hydration and swelling [55].

The combination of xanthan and locust bean gum (XlBG) was used for the controlled delivery of Metoprolol tartrate (MPT). Metoprolol tartrate is a nonselective beta-adrenergic blocking agent used for angina pectoris and treating hypertension and cardiovascular disorders. The drug MPT has a limitation related to bioavailability (30%) and drug releasing properties. The drug was suitable for a controlled drug release because it has a very short half-life of about 2–5 h. The rate of drug release was normal with zero-order kinetics and there is no chemical interaction between the drug and the used gum confirmed by DSC thermogram studies. The tablets formulated with xanthan and locust bean gum exhibited a sustained drug release due to the strong and elastic gel formed via the synergistic interaction between these two materials. The swelling and erosion studies revealed that the high ability to swell and the weight loss from the tablet was around 70% for 8 h [56]. *Konjac glucomannan* (KGM), isolated from the tubers of Amorphophallus konjac, was used for formulating sustained DDS with Xanthan. The small molecular drugs, theophylline and diltiazem, were used as the models. The KGM and Xanthan ratio of 1:1 was found to be suitable for obtaining formulation with physical integrity of the drugs and a sustained release of 8 h. In this, the diffusion coefficient value of KGM/XG was lower than individual KGMs, and the value close to XG indicates that the mobility of the drug is determined by the structure of XG. The synergistic interaction of KGM with Xanthan is responsible for the controlled drug release [57].

Mucilage from *Mimosa pudica* seeds contains d-xylose and d-glucuronic acid. These seeds are known to easily undergo hydration and swelling. Diclofenac was formulated with *Mimosa pudica* seeds and dibasic calcium phosphate (diluent). The resultant granules were studied for drug release in an aqueous dissolution media (pH 6.8, 37 °C) for 24 h. Additionally, the mucilage content and swelling property increased in the drug, and the erosion decreased. In response to the increase in the concentration of *Mimosa pudica* seeds, the drug release decreased. As a result, the drug release was dependent on the ratio of mucilage to drug. Granules with high mucilage content followed the diffusion type release while lower mucilage content led to a drug release via erosion and diffusion. Granules with a 1:40 mucilage to diclofenac ratio matches the commercially sustained release formulation [58]. Ganesh et al. used cashew nut tree gum for the sustained release of diclofenac Sodium. The tablets were formulated using cashew nut tree gum, hydroxypropyl methyl cellulose, and Carbopol. These tablets exhibited a sustained release. That is, the kinetics studies reveals that the drug releasing rate decreased as the CG content increased about 40% in the matrix. Since the gum content is too low, the formation of the gel layer did not occur on the surface. Higher amounts of CG lead to the prolonged drug release in a controlled manner. The advantages are lower dose intake, minimum blood level oscillations, low risk of adverse effects, and improved patient compliance [59].

When mixed with natural gums, the commercial xanthan gum proved to exhibit a synergistic effect for a colon-specific sustained release in a controlled drug delivery. The mixture of Xanthan and the natural gum galactomannan extracted from *Gleditsia sinensis Lam* was studied for the sustained release of theophylline. The formulation with 10% of each component released ~90% of the drug after 24 h. The synergistic interactions between these gums and the relaxational and diffusional drug release mechanism effectively controlled the drug release. The relation between hydration and drug release was characterized by the morphological changes and radial swelling behavior of the drug. Xanthan and galactomannan, with a ratio of 7:3, demonstrated a good performance in terms of the sustained release (75.5% after 24 h). These kinds of studies are evident for the potential of xanthan gum towards sustained and targeted DDS [60]. Mucoadhesive microspheres formulated with non-toxic and edible polysaccharide were extracted from the okra fruit (*Abelmoschus esculentus*). *A. esculentus* contains galacturonic acid, galctose, and rhamnose, where the components act as high absorbents due to its high-level polar groups. This leads to the formation complex with gel consistency and a bioadhesive compound. This formulation was studied for the nasal delivery of rizatriptan benzoate. This drug is used for narrowing brain blood vessels and treating severe headaches. Microspores were prepared via emulsification. Epichlorohydrin was used as the cross-linking agent. About 50% of the drug was released within 15 min and the remaining 50% was sustained for an hour. An increase in the polysaccharide concentration decreased the drug release. When administered to the nasal cavity of rabbits, the nasal retention of these microspheres was found to be better than the aqueous solution. The use of okra polysaccharides as a carrier for nasal delivery formulation improved the retention at the nasal surface and minimized the drainage via mucociliary clearance [61].

The mucoadhesive formulation-based oral DDS is suitable for sustained drug release. The good drug adhesion of DDS to the biological surface increases drug residence time and bioavailability. Mucoadhesive beads containing a glibenclamide *Plantago ovata F*. husk mucilage–alginate was prepared using a ionotropic gelation method and studied for sustained release. CaCl_2_ was used as the cross-linker. The drug encapsulation efficiency of the mucoadhesive beads was 94.43 ± 4.80% *w*/*w* with a rate of zero-order kinetics. Good mucoadhesive with the biological membrane was observed, and drug release was sustained for 10 h. The adhesive nature of beads was different for different pHs, with the gastric pH (1.2) being 64.88 ± 5.06%, but the intestinal pH was 30.47 ± 3.86%. When orally administered to alloxan-induced diabetic rats, a significant antidiabetic effect was noticed for a prolonged period, with a rapid reduction in the blood glucose level within 2–3 h. Therefore, the glibenclamide-Plantago ovata F. husk mucilage–alginate formulation proved to be suitable for the sustained release of glibenclamide and for improving patient compliance in managing noninsulin-dependent diabetes [62]. With the objective of drug delivery to the colon, gum from *Grewia mollis Juss* stem bark was used to formulate Ibuprofen. Compression-coated Ibuprofen tablets were formed using powder and granules of this gum or hydroxypropyl methyl cellulose (HPMC). The drug release was studied under simulated gastro-intestinal fluids. The GC-coated tablets exhibited the prolonged half-life (8.66 h) and the oral relative bioavailability of GC was noted as 87.65%, which indicates the remarkable reduced side effects and considered colon delivery system. Ibuprofen remained for a longer time in the gum powder-coated tablets than in others. In the in vivo studies conducted with New Zealand rabbits, it was confirmed that the gum powder-coated formulation delivered the drug to the colon, and the release in the gastrolienal tract was minimized. Thus, the Grewia mollis stem bark gum–Ibuprofen formulation can be effectively used in colon-specific DDS [63].

Almond gum (prunus amygdalus exudes) was used for the colon-specific formulation of 5-aminosalicylic acid, an anti-inflammatory agent known to be degraded by gastric fluid in stomach. Microcrystalline cellulose was used as diluents. The developed matrix was evaluated by various studies concerning diameter, hardness, weight variation, friability, and in vitro drug release. The in vitro drug release studies confirmed that at pH 1.2, the drug release was controlled, and after 10 h, the drug release was 50% [64]. Chitosan extracted from discarded shells of Carinosquilla multicarinata was used for the sustained release of Diclofenac. The Chitosan and tripolyphosphate formulation with the ratio of 2.5:1 exhibited maximum drug encapsulation efficiency. When studied using simulated biological fluid (pH 7.4), 10 h was taken to observe an 80% drug release. The drug release profile suggests that chitosan NPs are stable enough to deliver a sustained drug release than the free drug. This work proves that the marine scrap from fisheries can be effectively used in DDS meant for curing anti–inflammatory diseases [65].

Aloe vera (AV) mucilage-polyacrylamide DDS was prepared by polymerizing acrylamide using the free radical polymerization of acrylamide in the presence of Aloe vera mucilage. An increase in the concentration of AV mucilage enhanced swelling, porosity, and drug release, and meanwhile, decreased gel formation. High hydroxyl group content made this DDS more hydrophilic. In the case of DDS with 2% AV mucilage, within 30 min, the drug release exceeded 85% at pH 1.2 and pH 7.4. Mixing with hydrophobic type MGs can render a prolonged drug release property [66]. The buoyancy and anti-ulcer activity of okra mucilage was utilized for gastro-retentive DDS formulations for delivering antacids. This formulation minimized the side effects. As it is an anti-diabetic food, the use of okra has an additional advantage. The use of food products for DDS purposes can be useful to avoid a series of complex medical trials [67].

The super disintegrant property of MGs can be used for developing rapid DDS. Mucilage extracted from *Plantago ovata* (PO) was used to develop fast disintegrating Diclofenac potassium tablets. *Plantago ovata* mucilage is known to be a natural super disintegrant. Diclofenac Potassium is a non-steroidal, anti-inflammatory, analgesic, and antipyretic drug. It is absorbed faster in the gastrointestinal tract than by the Diclofenac sodium salt. PO mucilage-containing tablets exhibited fast disintegration (18 s) than the commercial disintegrant-containing (Sodium Starch Glycolate = 39 s, and Ac-Di-Sol = 49 s) tablets [68]. Apart from PO, a large number of MGs are available and studied for super disintegrant application. MGs from fenugreek seeds, Lepidium sativum, karaya gum, Cassia fistula, guar gum, *Hibiscus rosa-sinensis* Linn, pectin from mango peel, soy polysaccharides, dehydrated banana powder, and locust beans have been explored as super disintegrants in DDS [69].

## 6. Natural Source-Derived Exosomes in Drug Delivery Systems

**Figure 7:**
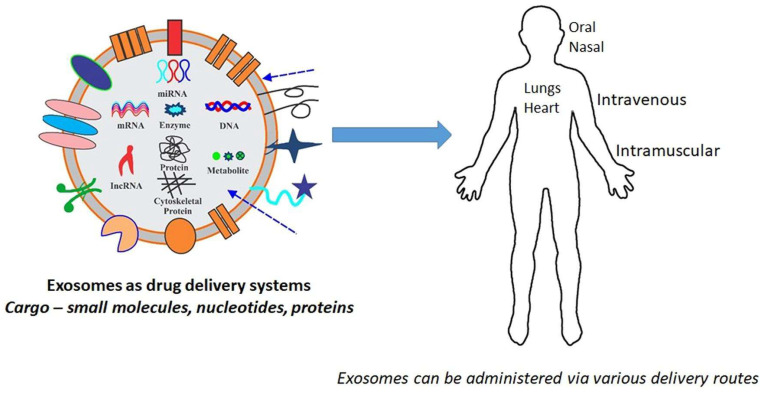
Application of Exosomes in DDS (adopted with permission from Ref. [72]).

As an efficient drug cargo, exosomes can be diversely modified with therapeutic biomolecules, nucleic acids, proteins, etc., which are critical in the discovery of advanced clinical diagnostics predominantly as drug delivery agents and biomarkers. Apart from exosomes, more extensive research has been established in the development of polymeric nanoparticles and liposomes for their broad range of applicability for antifungal and anticancer drugs, analgesics, and biocompatibility with a long-circulating capability by evading the immune system [70,71]. However, the intrinsic targeting ability of exosomes with minimally invasive effects to the cells or tissues recognizes them as a protected natural carrier bypassing immune responses and engulfment by lysosomes and phagocytosis. Figure 7 depicts the application of exomes in DDS.

Recently, exosomes have gained greater attention for their crucial role in bridging cell-to-cell communication during several pathophysiological processes. Although the exosomes were initially discovered in mammalian systems, its current spotlight as new biologically active substances in plants can extend their applicability as cargos for biomolecules that directly influences the growth of plants and develops protective mechanisms against pathogens [73]. Such plant exosomes are nanosized membrane-bound vesicles secreted by the plant cells, which serve as natural cargos for the transport of diverse biomolecules, including proteins, nucleic acids, lipids, and metabolites. To expand the scope of drug therapies, plant-derived exosome-like nanoparticles (PENs) are expected to boom as modern therapeutic modalities which are biocompatible, eco-friendly, and cost-effective with abundantly available nanoplatforms [72].

Generally, PENs undergo few common techniques such as biogenesis, isolation, sequential characterization, and other drug delivery methods that are necessary to enhance its stabilized utility, transport, and long-term circulation [74]. Based on the fundamental features and bioactivities of PENs, these exosomes and other secondary metabolites were widely extracted from diverse plant species including ginseng, grape, grapefruit, green tea, lemon, broccoli, etc., refs. [73,75]. Interestingly, plant exosomes are derived from the endosomal system through the biogenesis process involved in the formation of the intraluminal vesicles (ILVs) within the multivesicular bodies (MVBs) governed by endosomal sorting complex, which is required for transport (ESCRT) machinery. Two main pathways contribute to plant exosome biogenesis: the ESCRT-dependent pathway and the ESCRT-independent pathway. The ESCRT-dependent pathway facilitates the invagination of the endosomal membrane and cargo sorting, which plays a crucial role in the formation of ILVs and the subsequent exosome release. Meanwhile, the ESCRT-independent pathway involves lipid raft formation and exosome secretion (with the help of ESCRT-associated proteins) [76,77].

Intriguingly, the exosome release in certain plants, like grapefruit and tomato, are influenced by specific cargo compositions such as proteins, nucleic acids, lipids, and metabolites, which can be isolated from their flesh, juice extracts, and roots. Grapefruit and tomato exosome vesicles are known to contain diverse arrays of proteins, such as the enzymes that are involved in metabolic pathways (hydrolases, transferases, and oxidoreductases) the intercellular signaling molecules, including growth factors, cytokines, antioxidants (catalase and superoxide dismutase) protecting the cells from oxidative stress, and other flavor-related proteins [78,79]. Recently, natural exosome-like nanovesicles were designed (size: 131 nm) from edible tea flowers which remarkably triggered cellular apoptosis of breast tumors and inhibited lung metastasis conditions [80]. Such nanovehicles comprising large amounts of polyphenols, flavonoids, lipids, etc., exhibited strong cytotoxicity against the cancer cells, causing mitochondrial damage and arresting the cell cycle due to reactive oxygen species amplification. In addition, some reports utilized edible plant-derived exosomal NPs as potentially advanced therapeutic agents, such as ginger exosome-like NPs (GELNs) from the periodontal pathogen *Porphyromonas gingivalis*, for treating chronic periodontitis [70]; ginseng-derived exosomes (G-Exos) to effectively stimulate the neural differentiation of bone marrow-derived mesenchymal stem cells (BMSCs) by transferring the incorporated miRNAs to BMSCs [81]; and Beta vulgaris extract (BEX) for its anticancer and proangiogenic effects [82].

Some of the citrus fruit varieties, specifically under the Rutaceae family, have high therapeutic values due to the innate presence of flavanone hesperidin with exceptional hepatoprotective properties triggered via diverse cellular mechanisms, including improved activity of heme oxygenase 1, antioxidant response elements following enzymatic and mon-enzymatic pathways, C-reactive proteins, etc., [83]. Such essential components obtained from citrus have also been used as anti-corpulence and anticancer drugs [84]. Furthermore, specific groups of secondary metabolites (furanocoumarins) isolated from grapefruits like *Citrus paradisi* possess superior anti-inflammatory and anti-cancerous properties promoting bone health and regeneration [85]. Mostly, these properties resulted from the specific functioning of flavonoids, phenolic acids, and polyphenols present in grapefruit extracts that can generate reactive oxygen species intrinsically and can eventually become beneficial in decreasing the risk factors associated in cardiovascular health issues and several other age-related neurodegenerative disorders [86,87,88]. Constructing unique designs of PENs can unravel the diverse functionalities of plant exosomes holding control over the cellular and metabolic activities, eventually driving the delivery of natural compounds to specific cell targets with enhanced efficacy. The ability to transversely penetrate through the bio membranes, have hyper compatibility, and be less prone to allergic natures is what makes plant-based exosomes more compact and prominent vehicles for drug delivery and targeted therapeutic treatments.

## 7. Other Natural Sources Derived Materials in Drug Delivery Systems

Other than metals, polymers, and quantum dots, various nanomaterials are also active in DDS, due to their versatile properties, such as cell targeting, good biocompatibility, and loading capacity. Bio-modified cell plasma membranes are applied to Nano vesicles in biomedical applications. Also, DNA-based hydrogels used in drug delivery has a property of bioactivity toward drug prevention from chemical and enzyme degradation. Highly programmable DNA hydrogels are potential carriers for various DDS, to achieve high performance systems for chemotherapy, immunotherapy, and gene therapy [89]. Like that, extra cellular vehicles (EVs) derived from milk has immunogenicity and are non-toxic, which is supported for use in drug carriers. It has the characteristics of prolonged drug releasing capacity and enhances the cellular and reducing toxic effect. Recently, researchers have reported that milk EVs have increased anti-proliferative activities, as to determine from milk EVs certain encapsulated drugs such as berry anthocyanidins, paclitaxel, and aglycons [90]. The anti-proliferative effects of exomes were analyzed in various cancer cell lines, namely lung (A549, H1299), breast cancer cells (MCF7, MDA-MB-231), pancreatic (Panc1, DU145), ovarian, and colon cell lines (OVCA432, HCT116). The results showed that the inhibition of cell growth recorded in a 4–60-fold decrease when treated with 50 µM of Exo-Anthose for 72 h [91].

Another main biosource is lipid-based nanoparticles (L-NPs), which serves as nano carriers for drug delivery. The special characteristics of L-NPs are its potential to cross the gastrointestinal barrier and its great lipophilicity. As a result, L-NPs acts as a nano vesicle for less soluble drugs. There are many sources for L-NPs, such as fatty acids, glycerides, and phospholipids [92]. Rahila et al. reported an embelin-loaded phospholipid complex (EPC) containing embelin, which is a drug naturally occurring from the fruit of Emblia ribes. This drug is used as an analgesic, antimicrobial, antioxidant, and antitumor agent. The poor water solubility of this drug can be increased by combining it with phospholipids. In a deionized water medium, after 2 h, the free embelin shows a 19% drug release, and EPC attained a 99.80% drug release [93]. To this extent, the ginger derived L-NPs (GDLNPs) applied as lipid vehicles for siRNA were achieved via GDLNPs loaded with siRNA-CD. GDLPs have certain components such as phosphatidic acid, monogalactosyldiacyglycerol, and digalactosyldiacyglycerol, with 41.9%, 18,9%, and 27.4% of total lipids, respectively. The mono and digalactosyldiacyglycerol played a major role as a stabilizer for drug-loaded LPNPs. In this, GDLNPs/siRNA efficiently and selectively act against the colonic CD98 cells and reduce the gene expression by 20.2 ± 5.1% for 24 h of colonic CD98 (Colon-26 tumors). These LNPs shows a potential delivery of siRNA, which is equivalent to the Lipofectamine 2000 commercial drug delivery system. As shown in the results, the naturally derived GDLNPs show effective transfection results determined by the in vitro studies on various cell lines [94]. Similarly, the GDLNPs are used as drug carriers for doxorubicin (Dox) to Colon-26 tumors. The viability and apoptosis assay of GDLNPs exhibit good biocompatibility, reduced cell proliferation, and increased apoptosis at a concentration of 200 µM/L. Additionally, it shows an excellent targeted pH-dependent drug release to colon-26 tumors and an enhanced inhibition of tumor growth as compared with the free drug [95]. Ginger-derived L-NPs are widely used in targeted DDS chemotherapeutic agents, as well as delivering proteins to various types of cells due to its biocompatibility and the fact that it did not generate immune toxicity [96,97].

Another emerging bio-based NMs are cell membrane derived cargos, which vide various advantages such as the functional proteins on their surface and the natural cell-to-cell interactions. For the improvement of the activity in the anti-cancer drug, gemcitabine and paclitaxel were encapsulated with components derived from a pancreatic tumor cell membrane (CMNP-GEM-PTX). This result shows that the CMNP-GEM-PTX has high stability and a greater cytotoxicity effect on tumor cells (PANC-1). The efficiency of encapsulation for GEM is about 50% with a drug release capacity of 25% for 48 h. It did not reveal any immune toxicity effect or reduced adverse effect of treatments, and it will need clinical trials for further improvement [98]. Various cell membrane-based DDS attracted scientific attention. Cell membranes, such as the erythrocyte membrane, platelet membrane, leukocyte membrane, stem cell membrane, and cancer cell membrane, provide the ability to penetrate into tumor sites. These exhibited cell membrane-derived NMs encapsulated with therapeutic agents are considered as promising DDS, specifically for cancer therapy [99]. In this way, the adipocyte mesenchymal stem cell membrane (ADSC) loaded with NPs are used for treatment for rheumatic arthritis (RA), which is a chronic and inflammatory autoimmune disease. The in vitro and in vivo studies revealed that pro-inflammatory factors, such as IL-1β, IL-6, and tumor necrosis factor-α decreased, and the anti-inflammatory factor (IL-10) increased without causes of any adverse effects [100].

Smart nanocarrier-derived from viruses and bacteria have been applied as targeted or personalized DDS. The different types of viruses, such as adeno-associated viruses, lentivirus, and other animal viruses used various drugs as well as gene carriers and controlled the releasing of the therapeutic agent in DDS. Due to the potential physical stability of the viral particles, no chemicals or conditions affected the drug delivery process. In some cases, the viral coating plays a role in recognizing specific disease biomarkers or receptors and functions as triggerable agents. Even though there are still several challenges in therapeutic gene delivery, like purity, low production yield, target specificity, they need to be addressed [101]. Distinct natural source-based NMs for DDS applications are tabulated and presented in Table 1.

## 8. Conclusions and Outlook

The development of eco-friendly methodologies based on natural resources are inevitable for a sustainable future. The development of biomaterials has resulted in notable breakthroughs in the territory of medication delivery. These naturally sourced biomaterials provide generous benefits, including targeted medication delivery, controlled release, and improved therapeutic efficacy. Functional nanomaterials have changed drug administration using various methods, including nanoparticles, liposomes, dendrimers, and carbon-based nanomaterials, and they have the potential to overcome many of the drawbacks of traditional DDS. The functionalized NMs-enhanced solubility, stability, and enormous bioavailability makes them promising candidates for the therapeutic outcomes.

In addition, under certain circumstances, functional biomaterials can cross biological barriers, such as the blood–brain barrier, making it easier to distribute medication to previously inaccessible locations. The biocompatibility and safety profiles of these biomaterials are being worked on, along with ways to synthesize them more efficiently and upscale them for mass manufacturing. Efforts are also being made to deal with regulatory issues and guarantee the clinical application of these novel DDS. Natural resource-based DDS was found to be capable of improving treatment outcomes, lowering side effects, and enabling individualized medicine for effective healthcare.

The findings also indicate that the obstacles related to natural resource-based DDS need to be resolved. Microbes, plants extract, fruit, and other food source-based methods were developed for synthesizing NMs relevant to DDS. Food resource-based methods can create distress over the use of limited food resources for industrial purpose as it raises concern over the issue of feeding billions of people. The other issues of food-based materials are they are abundant in all seasons and require laborious extraction and purification processes. In countries like India, the annual vegetable and fruit wastage was estimated to be 18–40%. Therefore, the food waste-based method will be beneficial. Thus, in the future, agriculture and biowaste-based green synthesis methods must be given importance for developing on the industrial scale. The food waste biomass can also be converted into NCDs. The continuous availability of biowaste is useful for economic and large-scale production of NMs associated with DDS.

The synthesis of naturally derived NMs remains largely complicated, especially concerning the polymeric NPs. A detailed study may be required to address concerns about the allergens or toxic impurities found in the plant extracts. Moreover, there is no standard, optimized, and reproducible procedure established for the purpose of preparing the natural extract-based synthesis of metal NPs and NCDs. The addition of external pH stabilizing agents affects the eco-friendliness of the system, and the reaction control is often optimized by tuning the reaction temperature optimization, concentration, and extent of the bio-extract addition (volume/min). More attention needs to be made towards establishing a standard optimized procedure, at least for each type of plant extract meant to be used for the synthesis of metallic NMs. Though there is an endless source of natural resources, their performance largely remained inferior to their synthetic counterparts. The questions relevant to the manufacturing process and their actual impact on climate, human health, and the environment still need to be resolved. Aside from that, the entire life cycle of NMs, from biosynthesis to disposal, is not clearly established. Although there are still obstacles that need to be solved, the tremendous advancements in this area lay a solid groundwork for a next-generation drug delivery system.

## Figures and Tables

**Table 1:** Natural source-mediated nano materials application in DDS.

Nanomaterials	Natural Sources	Shape/Size (nm)	Drug Delivery	Reference
AgNPs	Horse chestnut	50 ± 5	Resveratol	[27]
Au, Ag, CdSe, EuSe NPs	Aliginate gel	Spherical, 21.6 ± 1.8, 14.1 ± 3.4, 33.1 ± 7.0, 23.1 ± 7.3	Doxorubicin, Rifampicin	[29]
AuNPs	Walnut tree bark	43 ± 2.2	Zonisamide	[30]
AuNCs	Green tea	Flower shape, 50	Doxorubicin	[32]
FeSeNPs	Protein of *Peseudomonus pudida* metallothionin	Spherical, 201.8 ± 4.7	Doxorubicin	[33]
AgNPs	*Poe annua*	Spherical, 36–43	Anti-cancer drug	[34]
Biopolymer- ZnO	Kappa carrageenan	Hexagonal, 97.03 ± 9.05	Used as biofilm	[38]
Membrane	Natural rubber latex	Pore size 2000, 6000, 10,000 pore/cm^2^	Ciprofloxacin	[40]
Molecularly Imprinted Polymer	Chitoson	-	Riboflavin	[41]
Carbon dots (CD)	Pasteurized milk	2.5 ± 1.0	LisinoPril	[50]
Quantum dots (QDs)	Mulberry leaves	2–4	Lycorine	[51]
N-CD	Korean Red Ginseng	13.94 ± 1.13	Rutin	[53]
Natural gum	*Geleditisia sinensis Lam*	-	Theophylline	[62]
Polysaccharide	Okra fruit	Microsphere	Rizhatriptan benzoate	[63]
Gum	Grewia mollis Juss stem bark		Ibuprofen	[64]
Chitosan	Shells of *Carinosquilla multicarniata*	-	5-amino salicylic acid	[66]
Exosome	Tea flower	131	Anti-cancer drug	[82]
Phospholipid	Soya	50–500	Embeline herbal drug	[95]
Polymer NPs	Cinnamon biopolymer	95.35 ± 10.25	Doxorubicin–Erlotinib	[102]

## Data Availability

Data is contained within the article.
